# Static High Voltage Actuation of Piezoelectric AlN and AlScN Based Scanning Micromirrors

**DOI:** 10.3390/mi13040625

**Published:** 2022-04-15

**Authors:** Chris Stoeckel, Katja Meinel, Marcel Melzer, Agnė Žukauskaitė, Sven Zimmermann, Roman Forke, Karla Hiller, Harald Kuhn

**Affiliations:** 1 Fraunhofer Institute for Electronic Nano Systems ENAS, 09126 Chemnitz, Germany; sven.zimmermann@zfm.tu-chemnitz.de (S.Z.); roman.forke@enas.fraunhofer.de (R.F.); karla.hiller@zfm.tu-chemnitz.de (K.H.); harald.kuhn@enas.fraunhofer.de (H.K.); 2 Center for Microtechnologies, Chemnitz University of Technology, 09111 Chemnitz, Germany; katja.meinel@zfm.tu-chemnitz.de (K.M.); marcel.melzer@zfm.tu-chemnitz.de (M.M.); 3 Fraunhofer Institute for Organic Electronics, Electron Beam and Plasma Technology FEP, 01277 Dresden, Germany; agne.zukauskaite@fep.fraunhofer.de

**Keywords:** AlN, AlScN, aluminum nitride, aluminum scandium nitride, micromirror, microscanner, piezoelectric

## Abstract

Piezoelectric micromirrors with aluminum nitride (AlN) and aluminum scandium nitride (Al_0.68_Sc_0.32_N) are presented and compared regarding their static deflection. Two chip designs with 2 × 3 mm^2^ (Design 1) and 4 × 6 mm^2^ (Design 2) footprint with 600 nm AlN or 2000 nm Al_0.68_Sc_0.32_N as piezoelectric transducer material are investigated. The chip with Design 1 and Al_0.68_Sc_0.32_N has a resonance frequency of 1.8 kHz and a static scan angle of 38.4° at 400 V DC was measured. Design 2 has its resonance at 2.1 kHz. The maximum static scan angle is 55.6° at 220 V DC, which is the maximum deflection measurable with the experimental setup. The static deflection per electric field is increased by a factor of 10, due to the optimization of the design and the research and development of high-performance piezoelectric transducer materials with large piezoelectric coefficient and high electrical breakthrough voltage.

## 1. Introduction

Micromirrors as scanning devices are reported intensively in literature with different electromechanical transducer principles. They are mostly classified into the electrostatic, electrothermal, electromagnetic, and piezoelectric micromirrors [1,2,3,4,5]. The piezoelectric transducer principle offers the advantages of high deflections at moderate excitation voltages and high dynamic ranges. Furthermore, a high degree of miniaturization and the monolithic integration of actuators and sensor elements is possible. In addition to the commonly used transducer material, lead zirconate titanate (PZT), piezoelectric AlN, and AlScN thin films can alternatively be used as piezoelectric transducers for actuation. Since 2018, several AlN and AlScN-based micromirrors have been presented. Shao et al. [6] presented the first AlN-based micromirror. The microsystem with a 0.2 × 0.2 mm^2^ mirror plate area and L-shaped bending actuators reached a resonant scan angle of 4° at 5 V and 63.3 kHz. Since then, further publications on AlN-based micromirrors have followed. Since June 2019, our preliminary work [7,8] includes resonantly operated 1D micromirrors with a 600 nm AlN film, a mirror plate length of 0.8 mm, and a chip size of 2 × 3 mm^2^. Large scan angles of up to 137.9° at 20 V and 3.4 kHz were reached in air. In October 2020, two 2D micromirror designs with a footprint of 2 × 2 mm^2^ and mirror plate diameter of 0.7 mm were developed to realize Lissajous and spiral scan trajectories [9]. For the Lissajous scanning design, a scan angle of 92.4° at 12,060 Hz and 123.9° at 13,145 Hz was reached at 50 V for the x- and y-axis, respectively. The spiral scanning design reached a scan angle of 91.2° at 13,834 Hz and 50 V. In 2021, a 2D circular-scanning AlN-based mircomirror with a large aperture of 7 mm for laser material processing was published by Senger et al. [10]. In air, a scan angle of 5° is reached at 40 V and 1265 Hz. Due to the application, no large deflection angles were specified. In order to achieve higher deflections with larger mirror apertures, vacuum packaging is often used in literature. A wobbling mode AlN-scanner for automotive applications was published in October 2019 by Pensala et al. [11]. The microsystem with an aperture of 4 mm and 6.75 × 6.75 × 2 mm^3^ chip size reached a scan angle of 30° at 1 V and 1.6 kHz by the implementation of a vacuum package. In 2020, Senger et al. [12] also presented a vacuum-packaged AlN-based micromirror with a 5.5 mm aperture. A Lissajous scan pattern with 50° × 20° scan angle was realized.

The previously mentioned micromirrors are exclusively driven in resonance to achieve sufficiently large tilt angles. Resonance frequency deviations, caused by variations of the ambient conditions like mechanical vibration and temperature change or heating due to light losses during laser irradiation, lead to a change of the tilt angle and, finally, result in errors in image formation and reconstruction [4,13]. Therefore, a static or quasi-static working mode has many advantages in regards of the electronics and drivers. In March 2019, Gu-Stoppel et al. presented an AlScN-based quasi-static micromirror with mirror plate diameter of 0.8 mm [14]. The mirror plate is mounted onto a pillar, which is deflected by four actuators hidden beneath. A high static scan angle of 50° at 150 V_DC_ was achieved by this novel construction. The challenge with this concept is a complex manufacturing process, which includes different wafer bonding processes for the micromirror assembly.

In this work, the static deflection and high voltage performance of the Design 1 MOEMS in [8] is investigated. Additionally, a technology is developed using a 2 µm Al_0.68_Sc_0.32_N with high thickness as transducer material for a direct comparison of the performance of the MOEMS with higher piezoelectric coefficient. Furthermore, the design is optimized for a chip size with twice the length and width of MOEMS (Design 2) to identify the performance gain for different chip footprints and further increase the deflection. By reducing the silicon spring width in relation to previous designs, the stiffness is decreased, targeting a high deflection per voltage.

## 2. Design

**Figure 1:**
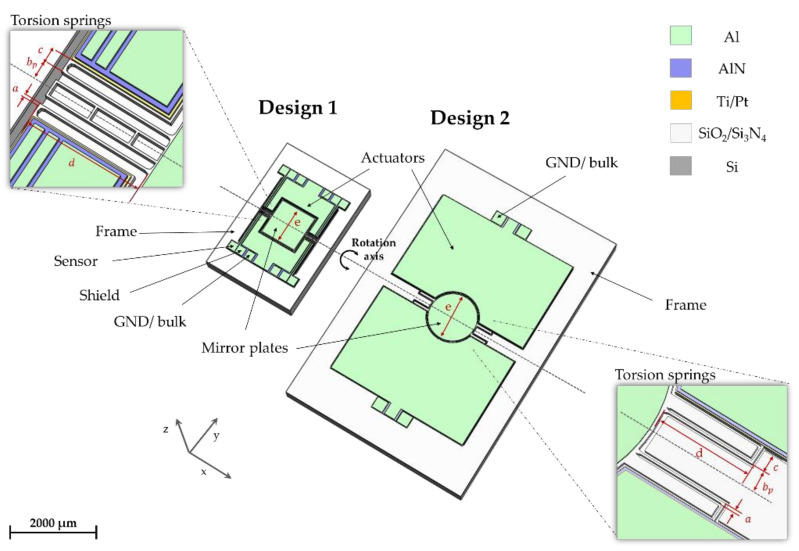
Schematic of the presented micromirror designs.

**Figure 2:**
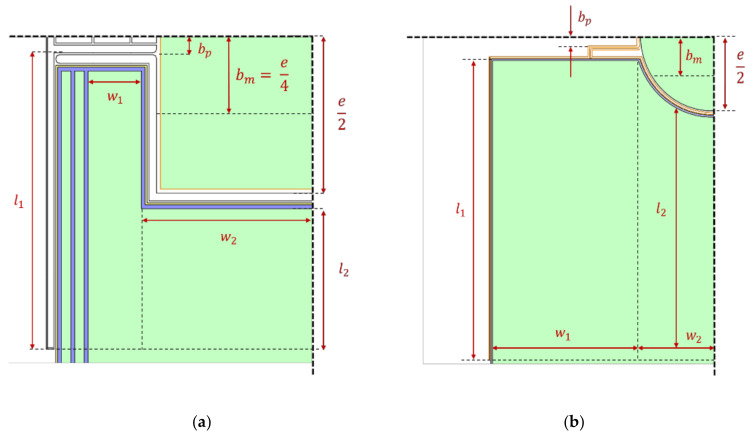
Quarter symmetrical model of the MOEMS: (**a**) Design 1; and (**b**) Design 2.

In Figure 1 the schematics of the fabricated Al(Sc)N micromirrors with 2 × 3 mm^2^ and 4 × 6 mm^2^ footprint are shown. In Table 1 the mirrors parameter are shown. The mirror plate is connected with two actuators by four L-shaped springs. The design and FEA of the 2 × 3 mm^2^ MOEMS is shown by Meinel et al. [8]. The MOEMS design and FEA process for optimization of the leverage effect is described by Meinel et al. [7] previously. In this publication, the static deflections of the MOEMS are calculated analytically. The parameters for the analytical calculation are given in Table 1. The actuators are described in a quarter symmetrical model of the MOEMS (Figure 2). The piezoelectric unimorphs are divided into two separate actuators in parallel. The actuator has the length l and the width w. Both actuators have a free displacement $\xi_{0}$ and a blocking force $F_{0}$.

(1) $$\xi_{0}=-3\cdot \frac{d_{31}\cdot l^{2}}{t_{p}^{2}}\cdot \frac{AB\cdot \left(B+1\right)}{D}\cdot V$$

(2) $$F_{0}=-\frac{3}{4}\cdot t_{p}\cdot E_{p\;}\cdot d_{31}\cdot \frac{AB\cdot \left(B+1\right)}{AB+1}\cdot \left(\frac{w_{1}\;}{l_{1}}+\frac{w_{2}\;}{l_{2}}\right)\cdot n\cdot V$$

(3) $$A=\frac{E_{S}}{E_{p}}\;;\;\;B=\frac{t_{S}}{t_{p}}\;;\;\;D=A^{2}\cdot B^{4}+2A\cdot \left(2B+3B^{2}+2B^{3}\right)+1$$

The free displacement is defined by the deflection of the longest actuator in the system. The blocking force is a sum of all four actuators n of the system. The actuator force in relation to the displacement at the leverage arm $\xi_{p}$ can be described as $F_{p}\left(\xi_{p}\right)$:

(4) $$F_{p}\left(\xi_{p}\right)=-\frac{F_{0}}{\xi_{0}}\cdot \xi_{p}+F_{0}$$

The microsystem has a resonance frequency as a result of the systems stiffness C and mass m. The mass is approximated as the mirror plate mass only. The stiffness is calculated by the resonance frequency f of the system. The force in relation to the stiffness and the deflection at the center of the mass $\xi_{m}$ can be described as $F_{c}\left(\xi_{m}\right)$.

(5) $$F_{c}\left(\xi_{m}\right)=\;C\cdot \xi_{m}=4\pi^{2}\cdot f^{2}\cdot m\cdot \xi_{m}$$

The relation between $\xi_{m}$ and $\xi_{p}$ is defined by the leverage arm distance to the center $b_{p}$ and the distance to the area center $b_{m}$:

(6) $$\xi_{m}=\frac{b_{m}}{b_{p}}\cdot \xi_{p}$$

The momentum of the actuators and the systems stiffness are in an equilibrium:

(7) $$M_{p}={\;M}_{m}$$

(8) $$F_{p}\cdot b_{p}=F_{c}\cdot b_{m}\;$$

(9) $$\left(-\frac{F_{0}}{\xi_{0}}\cdot \xi_{p}+F_{0}\right)\cdot b_{p}=\;C\cdot \frac{b_{m}}{b_{p}}\cdot \xi_{p}\cdot b_{m}\;$$

The deflection at the leverage arm position in relation to the leverage arm distance to the center of the MOEMS can be calculated with this equilibrium of the momentum:

(10) $$\xi_{p}\left(b_{p}\right)=\frac{F_{0}}{\frac{F_{0}}{\xi_{0}}-\frac{b_{m}^{2}}{b_{p}^{2}}\;\cdot \;C}$$

The deflection at the edge of the mirror plate $\xi_{e}$ in relation to the lever arm position $b_{p}$ is given by Equation (11).

(11) $$\xi_{e}\left(b_{p}\right)=\frac{F_{0}}{\frac{F_{0}}{\xi_{0}}+\frac{b_{m}^{2}}{b_{p}^{2}}\;\cdot \;C}\cdot \frac{b_{m}}{b_{p}}\cdot \frac{\frac{e}{2}}{b_{m}}=\frac{F_{0}}{\frac{F_{0}}{\xi_{0}}+\frac{b_{m}^{2}}{b_{p}^{2}}\;\cdot \;C}\cdot \frac{e}{2b_{p}}\;.\;$$

It should be noticed, that a LDV-based measurement of the deflection will not be done at the exact edge of the mirror plate, due to irregular reflections of the light. The measurement of the mirrors deflection in this paper is done in a distance to the mirror edge of approximately 50 µm. Additionally, the analytic equations do not include losses of the elastic energy, or mechanical stress and strain in the torsion springs.

**Figure 3:**
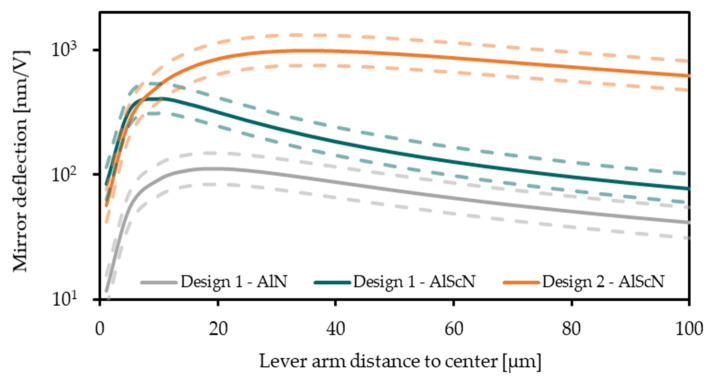
The static mirror plate deflection (mechanical tilt angle) per voltage in relation to the lever arm distance to the center is given for different designs and transducer materials. The parameter substrate height, piezoelectric charge coefficient, and resonance frequency are estimated with a 10% limit of variation. Therefore, an upper and lower limit of the approximated variation to the mechanical tilt angle is given.

In Figure 3 the mirror plate deflection is shown in relation to the lever arm distance to the center. An optimum lever arm length can be identified, depending on the systems mass and stiffness as well as the blocking force and free deflection of the actuators. The parameter of silicon height, piezoelectric charge coefficient, and resonance frequency are estimated within a 10% limit of variation. For Design 1 with AlN as transducer and a 40 µm lever arm distance the calculated deflection is in the range of 65 nm/V to 116 nm/V. Using Al_0.68_Sc_0.32_N and Design 1 increases the deflection up to 143 nm/V to 241 nm/V. Design 2 has a lever arm distance to the center of 80 µm. For this design and Al_0.68_Sc_0.32_N a deflection of 563 nm/V to 959 nm/V is calculated.

## 3. Fabrication

**Figure 4:**
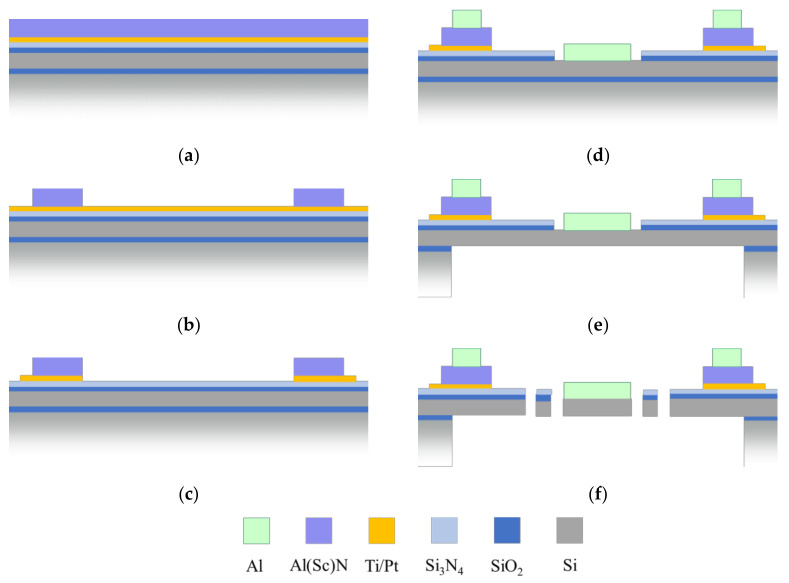
Fabrication process flow: (**a**) Initial layer stack; (**b**) AlN wet etching; (**c**) Pt structuring; (**d**) Dry etching of silicon nitride and wet etching of silicon oxide, aluminum deposition; (**e**) backside structuring by DRIE; and (**f**) dry etching of device silicon.

The wafers with AlN and Al_0.68_Sc_0.32_N piezoelectric layers are processed with an identical process flow and process parameters, except for the deposition and etching of the piezoelectric material. Figure 4 illustrates the device fabrication process flow. The microsystem fabrication is based on 150 mm SOI technology with 575 µm thick handle wafer and 20 µm device-silicon thickness. First, a 1 µm thermal oxide is grown by oxidation. LPCVD silicon nitride with a layer thickness of 100 nm is used as an isolation layer due to its selectivity against HF wet etching processes. The piezoelectric layer stack starts with a seed layer of 100 nm platinum. For a better adhesion with the substrate, a 20 nm titanium film is used. The Pt is deposited in <111> orientation to minimize the elastic energy to a <0002> Al(Sc)N crystal. A 600 nm AlN or 2000 nm Al_0.68_Sc_0.32_N, respectively, and a 100 nm PECVD SiO_2_ layer are deposited as piezoelectric material. Table 1 summarizes the PVD deposition conditions for the AlN and Al_0.68_Sc_0.32_N layer. DC magnetron process with an Al target (double ring magnetron target 120 mm and 123–236 mm, purity 5N5) in 100% nitrogen atmosphere is used for AlN. In the case of Al_0.68_Sc_0.32_N, co-sputtering from 5N5 Al and 4N pure Sc targets in pulsed DC mode in 100% nitrogen atmosphere is used at combined power of 1000 W. AlScN growth optimization, as well as structural and compositional analysis are discussed elsewhere [15,16]. 

The adhesion of photoresists during the AlN wet etch process is not sufficient. Therefore, a 100 nm SiO_2_ is the hard mask material for the patterning process. AlN and Al_0.68_Sc_0.32_N wet etching is done with 85% phosphoric acid solution (H_3_PO_4_) at 80 °C (Figure 4b). The etch rate of AlN is 1.4 nm/s. The Al_0.68_Sc_0.32_N has a etch rate of 6.7 nm/s. Test wafer with Al_0.86_Sc_0.14_N have etch rates of 4.2 nm/s. This indicates a correlation of higher etch rates and higher Scandium ratios in the piezoelectric transducer.

The platinum and titanium are structured via tungsten hard mask by a dry etch process (Figure 4c) which is monitored with an optical emission spectrometer. By analyzing the species in the plasma, an etch stop can be defined as soon as the Ti/Pt is etched and the dry etching of the silicon nitride starts. In Figure 4d, the silicon nitride is patterned by RIE and the silicon oxide is wet etched. This enables an aluminum deposition on a smooth silicon surface. The 800 nm aluminum layer serves as reflective layer on the mirror plate and as upper electrode for excitation of the piezoelectric actuators. After wet etching of the aluminum layer, the handle wafer silicon is structured by DRIE using the buried SiO_2_ of the initial SOI wafer as etch stop (Figure 4e).

By variation of the exposure parameters in the lithography as well as the DRIE parameters, a side wall shift of the silicon springs can be done to reduce the systems stiffness. This process can be done by using different resists, exposure times, or another DRIE process recipe. For the wafer with AlN this side wall shift is about 0.85 µm at each sidewall. This results in a change of the spring with from a = 5 µm to a′ = 3.3 µm. For the wafer with Al_0.68_Sc_0.32_N a side wall shift of 1.35 µm is used. The lower stiffness should result in higher deflection per voltage and further increase the MOEMS performance compared to systems with high resonance frequency.

## 4. Measurement Setup

By using a Polytec MSA 400 Laser-Doppler-Vibrometer (LDV) with OFV 5000 Controller, the frequency-response-functions (FRF) of the MOEMS are recorded. A chirp signal with an amplitude of ±1 V is applied to the top electrode of one actuator. The opposite actuator is driven with a 180° phase shifted signal with the same frequency and amplitude. So, the actuators work in antiphase mode. The amplitude of deflection is measured at the mirror plate edge. By measuring the resonance frequency, the stiffness of the MOEMS can be identified indirectly. This allows to compare static performance values for similar mechanical parameters of the MOEMS.

**Figure 5:**
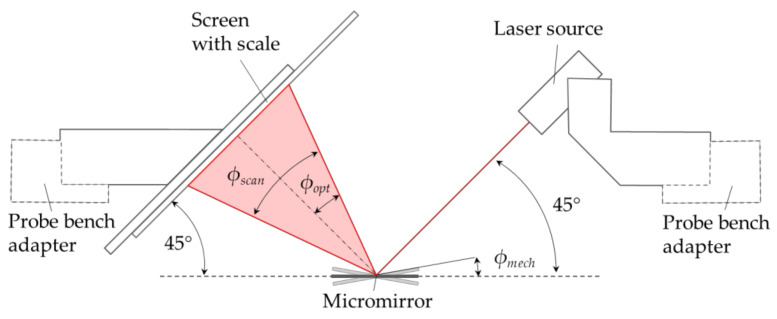
Schematic of the experimental setup [9].

For measuring higher tilt angles, a high-deflection setup was introduced in [8,9]. Mechanical tilt angles up to approximately 15° can be measured. A laser beam is projected onto the mirror at a 45° angle. The mirror reflects it on an adjustable screen with a metric scale, which is also attached at a 45° angle to the mirror. The components like the laser mount and screen are fixed on a ring, adapted to the prober station (see Figure 5).

The transversal piezoelectric coefficient defines the piezoelectric crystal deformation in result of an electric field. If samples with different piezoelectric material thicknesses and piezoelectric coefficients are used, the electric voltage as parameter for actuation is not sufficient to interpret the system performance. Therefore, the results are additionally documented in relation to the electric field in MV/m.

## 5. Results and Discussion

### 5.1. Frequency Response and Small Signal Actuation

**Figure 6:**
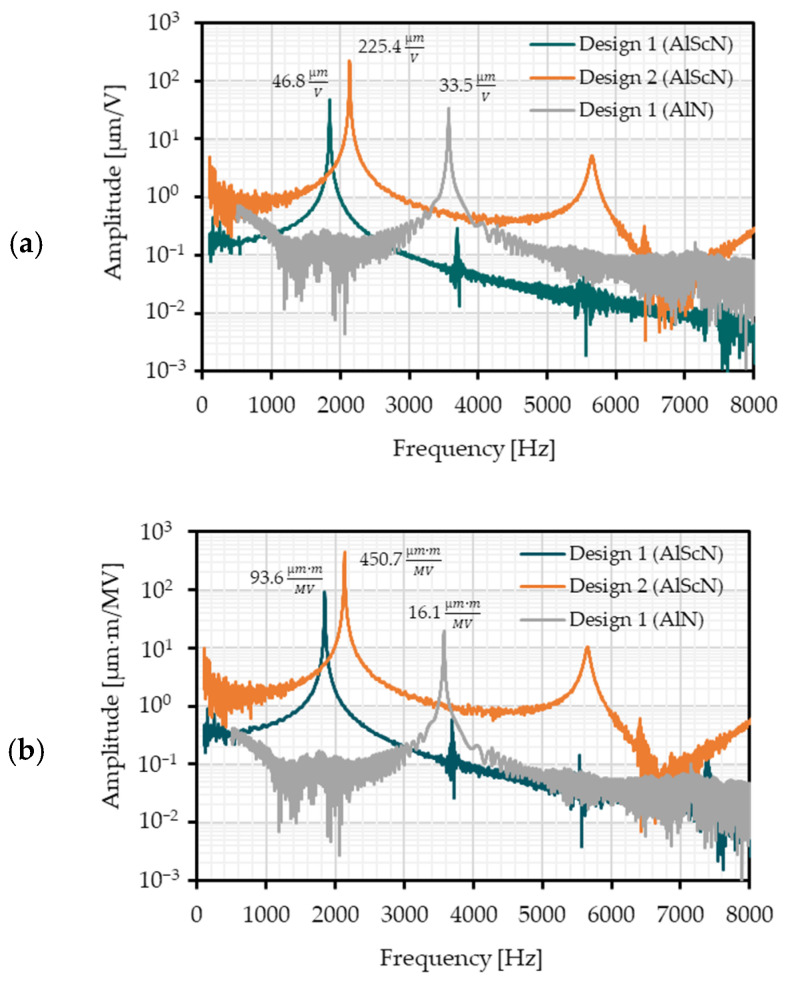
Frequency resonance functions of the presented micromirrors: (**a**) Mirror deflection per voltage; and (**b**) Mirror deflection per electric field.

**Figure 7:**
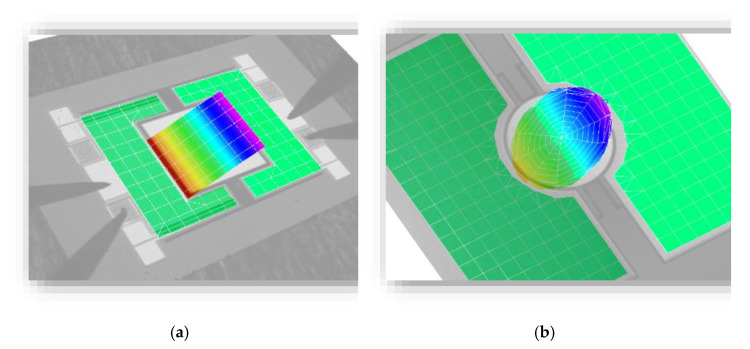
Motion scan images of exemplary micromirrors recorded by Laser-Doppler-Vibrometry: (**a**) Design 1; and (**b**) Design 2.

In Figure 6a,b the FRFs are depicted in logarithmic scaling. The amplitude is measured for a range of six decades. Due to the high deflection in resonance, the LDV sensor sensitivity is low. Therefore, there is a significant noise for low amplitudes. The measurement values are given in Table 2. A motion scan image of exemplary micromirrors of Design 1 and Design 2 in torsional mode is shown in Figure 7.

An analytical calculation is shown to describe the static deflection of the 1D MOEMS. For a MOEMS with AlN and Design 1 a static deflection of 65 nm/V up to 116 nm/V is calculated. The measured deflection is 61.1 nm/V. One reason for the smaller deflection in the manufactured system can be the loss of elastic energy in the torsion spring with a high stiffness, which is not modeled by the analytical formulas. The measured MOEMS deflection with Design 1 and Al_0.68_Sc_0.32_N is 157.6 nm/V. The measured deflection is within the calculated deflection range of 143 nm/V to 241 nm/V. For Design 2 with Al_0.68_Sc_0.32_N as piezoelectric transducer, the modeled deflection of 563 nm/V to 959 nm/V matches with the measurement result of 667.3 nm/V.

For Design 1, the AlN MOEMS has a resonance frequency of 3444 Hz. For the same design, the resonance frequency of the Al_0.68_Sc_0.32_N MOEMS is 1819 Hz. The side wall shift of the silicon results in a decrease of the resonance frequency and, therefore, in a lower stiffness of the system. The resonant mechanical tilt angle of the AlN based MOEMS (Design 1) is 2.8° at 1 MV/m. For the Al_0.68_Sc_0.32_N MOEMS an angle of 11.9° at 1 MV/m is measured. The deflection in relation to the electric field is increased by a factor of 4.

Due to the larger chip area and actuator length and width of Design 2, the deflection is increased to 35.6° per MV/m in resonance. The resonance frequency for the MOEMS with Design 2 is 2121 Hz. In total, the resonant deflection from a MOEMS with AlN and Design 1 compared to Al_0.68_Sc_0.32_N with Design 2 increased from 2.8° to 35.6° per 1 MV/m. This is a factor of 12.

### 5.2. Static High Voltage Actuation

**Figure 8:**
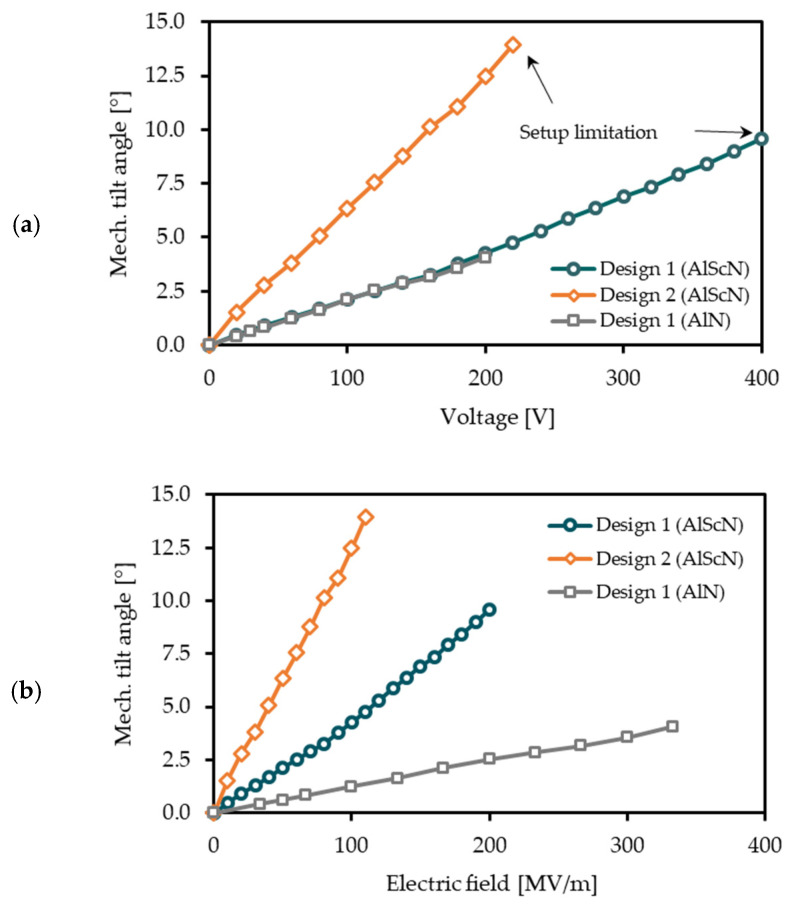
Performance of the presented micromirror designs at static high voltage actuation: (**a**) Mechanical tilt angle versus voltage; and (**b**) Mechanical tilt angle versus electrical field.

In this section, the scanning characteristics of three selected micromirror samples in torsional mode for voltages of up to 400 V are shown. 400 V is the maximum voltage of the power supply in the setup. Due to limitations in the measurement setup, deflections up to 15° mechanical deflection can be measured. In Table 3 and Figure 8 the static mechanical deflections of the systems are shown.

**Figure 9:**
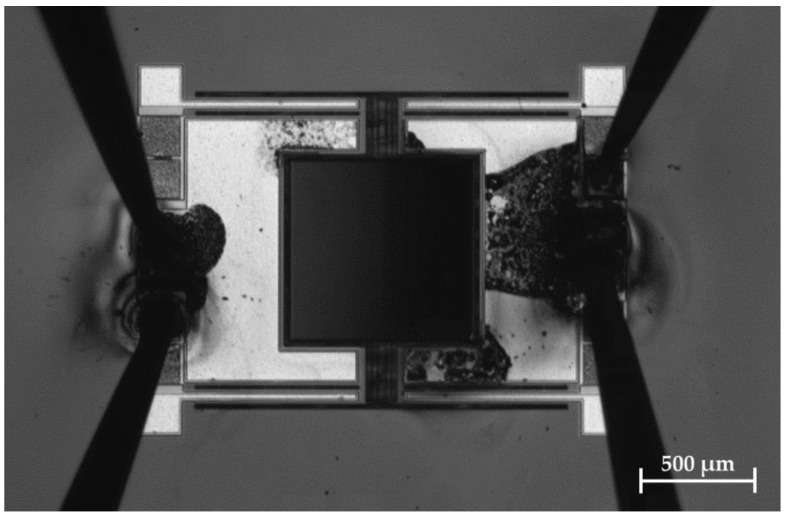
Photography of an exemplary micromirror of Design 1 with electric breakthroughs at 220 V. The electric contact of the MOEMS is done with micro needles on a probe station.

Electric breakthroughs are observed for AlN chips at voltages higher than 200 V (Figure 9). Up to 400 V actuation voltage is used for the samples with Al_0.68_Sc_0.32_N. The reason is the high electric field for the 600 nm thin AlN layers compared to the 2 µm thick Al_0.68_Sc_0.32_N. 

For Design 1 the maximum deflection is 9.6° at 400 V with Al_0.68_Sc_0.32_N as piezoelectric transducer. AlN-based chips show deflections up to 4.1° at 200 V. By comparing the deflection of the MOEMS in relation of the electric field, the use of 2 µm Al_0.68_Sc_0.32_N and lower system stiffness increased the static deflection by a factor of approximately 4. It can be assumed that the high piezoelectric coefficient of the Al_0.68_Sc_0.32_N is one major reason for the larger deflection. In addition, the lower stiffness of the Al_0.68_Sc_0.32_N based MOEMS influences the absolute and relative deflection and needs further investigations.

**Figure 10:**
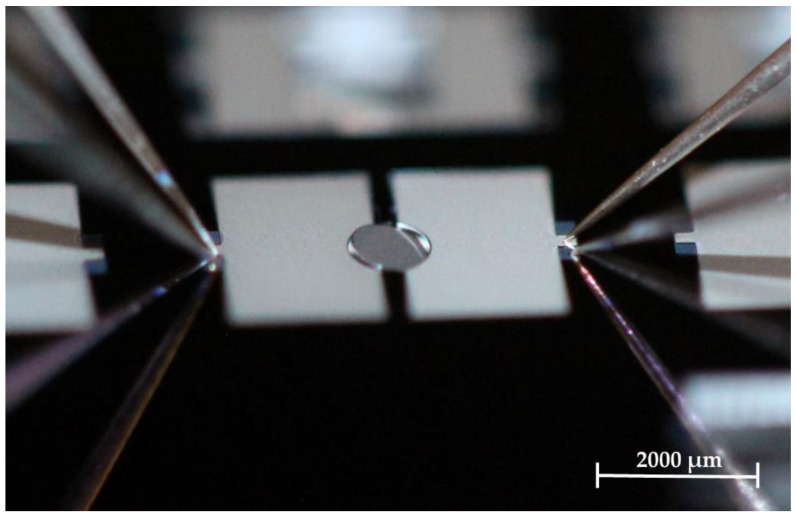
Photography of an exemplary micromirror of Design 2 in static operation (12.5°, 200 V). Captured by a single lens reflex (SLR) camera (Canon EOS 600D) and macro lens.

Design 2 enabled deflections of up to 13.9° at 220 V, which is the limit of the measurement setup. The deflection per electric field of Design 2 is increased by a factor of 3 in relation to Al_0.68_Sc_0.32_N-MOEMS with Design 1. Design 2 with Al_0.68_Sc_0.32_N has more than ten times of the deflection per electric field compared to the previously reported AlN based MOEMS with Design 1. The scan angle can be defined by four times the mechanical tilt angle. Therefore, scan angles up to 55.6° for static displaced Al_0.68_Sc_0.32_N MOEMS are shown. Figure 10 shows a photography of a static deflected MOEMS of Design 2 with Al_0.68_Sc_0.32_N at 200 V.

In Figure 8 the linearity for deflections < 15° can be seen. Relevant non-linear effects are not observed for the static deflections. Therefore, stress-stiffening effects have minor relevance for both MOEMS designs and static deflections < 15°.

For high electric fields the AlN and Al_0.68_Sc_0.32_N shows electric breakthroughs. In Figure 9 a chip is shown after a breakthrough. Optically, lightning discharges were observed spontaneously. If a lightning appears at one position of the chip, an avalanche effect starts immediately and multiple areas of the chip show electric breakthroughs. The positions of the breakthroughs are random. Therefore, imperfections of the AlN and Al_0.68_Sc_0.32_N growth could be the reason for the breakthrough. Nevertheless, a very electric field of up to 200 MV/m is applied to the piezoelectric layer, which indicates a high quality of the crystal growth and structure.

Future measurement setups need power supplies with voltages higher 400 V DC and a larger optical bank for the documentation of lager scan angles.

**Figure 11:**
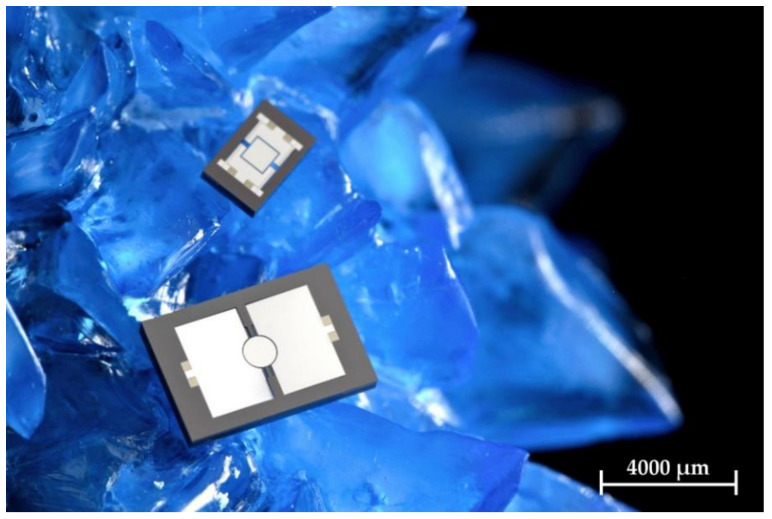
Photography of the Design 1 and Design 2 MOEMS with piezoelectric Al_0.68_Sc_0.32_N actuators on a copper sulfate crystal as a backdrop.

Figure 11 shows a photography of the Design 1 MOEMS with 2 × 3 mm^2^ footprint and the Design 2 MOEMS with 6 × 8 mm^2^ chip size in comparison.

## 6. Conclusions

The AlN and Al_0.68_Sc_0.32_N is was developed for the use at high electric fields up to 200 MV/m to increase the maximum electric energy the system can transform into a deflection. Additionally, the use of Al_0.68_Sc_0.32_N, in comparison to AlN, increased the static deflection per electric field by a factor of 3.5. This shows the impact of AlScN based transducer materials for piezoelectric microsystems. By using 2 µm thick Al_0.68_Sc_0.32_N with high electric breakdown voltage, the maximum actuation voltage was increased up to 400 V.

In Table 4 a comparison of the MOEMS Designs 1 and 2 with AlN and AlScN and the micromirror of Gu-Stoppel et al. [14] is presented. A figure of merit (FOM) is shown as a product of mirror diameter, respectively, mirror length, and scan angle. Another figure of merit considers the influence of the stiffness of the MOEMS by including the resonance frequency [1]. The presented MOEMS Design 1 and 2 with Al_0.68_Sc_0.32_N as transducer material have very high values for the FOMs. The FOM for the Design 2 MOEMS with Al_0.68_Sc_0.32_N is FOM = θ · e · f_res_ = 116.8 m·°·Hz and therefore 3.2 times higher than the reference in literature. The reason therefore can be a 2 µm thick Al_0.68_Sc_0.32_N with high piezoelectric coefficients and the use of high voltages as well as optimized design parameter with a leverage effect. However, Gu-Stoppel et al. [14] were able to manufacture a 2D MOEMS on a very small footprint using vertical silicon integration technologies. 

In summary, two different MOEMS designs with AlN and Al_0.68_Sc_0.32_N as piezoelectric actuator materials are compared. AlN and Al_0.68_Sc_0.32_N driven MOEMS with static scan angles up to 55.6° were fabricated. The chip performances for different designs and transducer materials with focus on the static actuation were compared. The use of Al_0.68_Sc_0.32_N, larger actuators, softer springs, the increased thickness of the transducer, and a material with high electrical breakdown voltages enabled the increase of the performance. The resonant deflection per electric field increased by a factor of 12. The static deflection per electric field increases more than 10 times due to the optimization in design and transducer material. The development of high-performance transducer materials and optimized MOEMS designs will allow miniaturized and robust micro optics with large static scan angles.

## Figures and Tables

**Table 1:** Comparison of the parameter of the micromirror designs and transducer materials.

	Symbol ^1^	Design 1 AlN	Design 1 AlScN	Design 2 AlScN
**General parameter**				
Piezoelectric layer		AlN	Al_0.68_Sc_0.32_N	Al_0.68_Sc_0.32_N
Thickness of piezoelectric layer [nm]	$t_{p}$	600	2000	2000
Electric field at 1 V [MV/m]		1.67	0.5	0.5
Silicon side wall shift [µm]		~0.85	~1.35	~1.35
**PVD parameter**				
Nitrogen concentration [%]		100	100	100
Pressure [Pa]		0.7	0.36	0.36
Substrate temperature [°C]		350	300	300
DC power [W]		2120	625 (Al) + 375 (Sc)	625 (Al) + 375 (Sc)
**Geometrical parameter**				
Spring width (mask) [µm]	a	5	5	7
Spring width (fabricated) [µm]	a′	~3.3	~2.3	~4.3
Lever arm distance to center [µm]	$b_{p}$	40	40	80
Distance to center of area [µm]	$b_{m}$	200	200	187.5
Lever arm distance to actuator [µm]	c	20	20	70
Spring length [µm]	d	255	255	1030
Mirror plate length/diameter [µm]	e	800	800	1000
Actuator width (quarter model) [µm]	$w_{1}$ $w_{2}$	140 440	140 440	1000 505
Actuator length (quarter model) [µm]	$l_{1}$ $l_{2}$	760 360	760 360	2020 1710
**Parameter for analytical calculation**				
Number of actuators in the model	n	4	4	4
Thickness silicon substrate [µm]	$t_{s}$	21 ± 10%	21 ± 10%	21 ± 10%
PE charge coefficient [pm/V]	$d_{31}$	−2 ± 10%	−5 ± 10%	−5 ± 10%
Resonance frequency [kHz]	f	3.5 ± 10%	2 ± 10%	2 ± 10%
E-Modulus of PE transducer [GPa]	$E_{p}$	108	108	108
E-Modulus of silicon [GPa]	$E_{s}$	63.9	63.9	63.9
Actuation voltage [V]	V	1.0	1.0	1.0
Mass of the mirror plate [ng]	*m*	31 ± 10%	31 ± 10%	38 ± 10%

^1^ Symbolism according to Figure 1 and Figure 2.

**Table 2:** LDV measurement results for the resonant and static deflections of the MOEMS Designs with different transducer materials.

Parameters ^1^		Design 1	Design 1	Design 2
		600 nm AlN	2000 nm Al_0.68_Sc_0.32_N	2000 nm Al_0.68_Sc_0.32_N
**(Quasi)static parameters**				
Mirror deflection (nm)				
	At 1 V	61.1	157.6	667.3
	At 1 MV/m	36.7	315.2	1334.6
Mech. tilt angle (m°)				
	At 1 V	8.8	22.6	76.5
	At 1 MV/m	5.3	45.1	152.9
**Resonant parameters**				
Resonance frequency (Hz)		3444	1819	2121
Mirror deflection (µm)				
	At 1 V	33.1	41.4	152.9
	At 1 MV/m	19.9	82.7	305.7
Resonant mech. tilt angle (°)				
	At 1 V	4.7	5.9	17.8
	At 1 MV/m	2.8	11.9	35.6

^1^ Parameters are medium values over five samples.

**Table 3:** Comparison of the static mechanical tilt angles of the several micromirror designs and piezoelectric transducer technologies.

Static Parameters		Design 1	Design 1	Design 2
		600 nm AlN	2000 nm Al_0.68_Sc_0.32_N	2000 nm Al_0.68_Sc_0.32_N
Mech. tilt angle (°)				
	At 100 V	2.1	2.1	6.3
	At 200 V	4.1	4.3	12.5
	At 400 V	—	9.6	— ^1^
	At 50 MV/m	0.6	2.1	6.3
	At 100 MV/m	1.2	4.3	12.5
	At 200 MV/m	2.5	9.6	— ^1^
Maximum mech. tilt angle (°)		4.1 (at 200 V)	9.6 (at 400 V)	13.9 ^1^ (at 220 V)

^1^ Limit of measurement setup.

**Table 4:** Comparison of (quasi-)static driven micromirrors based on piezoelectric AlN and AlScN of current literature and this work.

Specification	Unit	Ref. [14]	This Work		
			Design 1	Design 1	Design 2
Transducer material		AlScN	AlN	AlScN	AlScN
Material thickness	nm	1000	600	2000	2000
Mirror plate length (e)	mm	0.8	0.8	0.8	1.0
Chip size	mm²	1.4 × 1.4 ^2^	2 × 3	2 × 3	4 × 6
Res. frequency (f_res_)	kHz	0.9	3.4	1.8	2.1
Drive voltage	V_DC_	150	200	400	220
Scan angle (θ)	°	50	8.4	38.4	55.6 ^1^
FOM: θ · e	mm · °	40.0	6.7	30.7	55.6
FOM: θ · e · f_res_	m · ° · Hz	36.0	22.8	55.3	116.8

^1^ Limited by measurement setup. ^2^ Estimated chip size with frame.
